# Genome-Wide Analysis of the WOX Gene Family and Function Exploration of GmWOX18 in Soybean

**DOI:** 10.3390/plants8070215

**Published:** 2019-07-11

**Authors:** Qingnan Hao, Ling Zhang, Yanyan Yang, Zhihui Shan, Xin-an Zhou

**Affiliations:** 1 Oil Crops Research Institute of Chinese Academy of Agriculture Sciences, Wuhan 430062, China; 2 Chinese Academy of Agricultural Sciences/Key Laboratory for Biological Sciences of Oil Crops, Ministry of Agriculture, Wuhan 430062, China; 3 Jilin Provincial Key laboratory of Agricultural Biotechnology, Jilin Academy of Agricultural Sciences, Changchun, Jilin 130033, China

**Keywords:** soybean, homeobox genes, evolution, expression, function

## Abstract

WUSCHEL-related homeobox (WOX) is a family of transcription factors that are unique to plants and is characterized by the presence of a homeodomain. The WOX transcription factor plays an important role in regulating plant growth and development and the response to abiotic stress. Soybean is one of the most important oil crops worldwide. In this study, based on the available genome data of soybean, the WOX gene family was identified by bioinformatics analysis. The chromosome distribution, gene and protein structures, phylogenetic relationship and gene expression patterns of this family were comprehensively compared. The results showed that a total of 33 putative WOX genes in the soybean genome were found and then designated as GmWOX1- GmWOX33, which were distributed across 19 chromosomes except chromosome 16. Multiple sequence analysis of the GmWOX gene family revealed a highly conserved homeodomain. Phylogenetic tree analysis showed that 33 WOX genes could be divided into three major clades (modern/WUS, intermediate and ancient) in soybean. Of these 33 WOX genes, some showed differential expression patterns in the tested tissues (leaves, pods, unopen and open flowers, nodules, seed, roots, root hairs, stems, shoot apical meristems and shoot tips). In addition, the expression profile and qRT-PCR analysis showed that most of the GmWOX genes responded to different abiotic stress treatments (cold and drought). According to the expression pattern of GmWOX genes in the high regeneration capacity soybean material P3, overexpression of GmWOX18 was selected for function analysis. The overexpression of GmWOX18 increased the regeneration ability of clustered buds. The results will provide valuable information for further studies on the roles of WOX genes in regulating soybean growth, development and responses to abiotic stress, as well as a basis for the functional identification and analysis of WOX genes in soybean.

## 1. Introduction

The WUSCHEL-related homebox (WOX) gene family is a unique transcription factor in plants and belongs to the homeobox (HB) superfamily. Its members all contain homeodomain (HD) with 60–66 amino acid residues [1,2]. The specificity of the HD domain sequence makes the WOX gene family different from other HB families [3]. The homeodomain binds DNA through a helix-turn-helix (HTH) structure. The HTH motif is characterized by two α-helices, which make intimate contacts with the DNA and are joined by a short turn [3].

According to previous reports, WOX genes have a wide range of functions, including embryonic development, embryonic polarization, maintenance of meristematic stem cells, development of lateral organs, seed formation and regeneration of isolated tissues and organs. Fifteen WOX members, AtWUS and AtWOX1-AtWOX14, have been found in the Arabidopsis thaliana genome, and the functions of each gene have been studied clearly [3,4,5]. According to evolutionary relationships, WOX members can be classified into three evolutionary branches. They are the modern clade, the intermediate clade and the ancient clade [3,6,7]. WOXs of lower plants (green algae, non-vascular moss and Physcomitrella patens) belong only to the ancient clade, however those of higher plants are present in all three clades [3,8]. Earlier studies found that AtWUS promotes central identity in both indeterminate shoots and determinate floral meristems and plays an important role in maintaining their structural and functional integrity in Arabidopsis thaliana [5]. Overexpression of the WUS gene from Arabidopsis promotes somatic embryogenesis and induces organogenesis in cotton (*Gossypium hirsutum* L.) tissues cultured in vitro [9]. Furthermore, in the absence of exogenous hormones, overexpression of GhWUSs in Arabidopsis promoted shoot regeneration from excised roots [10]. AtWOX1 plays an important role in meristem development in Arabidopsis by regulating CLV3 expression or SAMDC activity [11]. AtWOX2 is required in the egg cell and zygote for early pre-embryo and cotyledon boundary formation during embryo development [4,12]. AtWOX3 is expressed at the margins of leaf and floral organ primordia for recruiting founder cells from all meristem layers to form lateral domains of both vegetative and floral organs [13]. As a homolog of AtWOX3, the maize NARROW SHEATH (NS) gene is involved in the growth of the leaf sheath and the proximal blade region [14]. OsWOX3, which is a homolog of AtWOX3, is required in the leaf and floral organ primordia in rice [15,16,17]. AtWOX4 was determined to be an essential regulator in auxin-dependent regulation of lateral plant growth (WOX4 imparts auxin responsiveness to cambium cells in *Arabidopsis thaliana*). AtWOX5 has a role in the root apical meristem as a negative trigger of IAA homeostatic mechanisms [18]. The PRETTY FEW SEEDS2 gene encodes a homeodomain protein (AtWOX6) that regulates ovule development [19]. An AtWOX6 named HOS9-1 is important for plant growth and development and plays a role in freezing tolerance by affecting the activity of genes that are independent of the CBF pathway [20]. AtWOX7 plays an important role in coupling the lateral root development program and sugar status in plants [21]. WOX2 and STPL/WOX8 function in promoting cotyledon boundary formation in Arabidopsis [22]. STIMPY/WOX9 identifies a new genetic pathway integrating developmental signals with cell-cycle control, and it is required for Arabidopsis meristem growth and maintenance [23]. WOX11 is an integrator of auxin and cytokinin signaling which feeds into RR2 to regulate cell proliferation during crown root development [24]. Some data indicate that the expression switch from WOX11/12 to WOX5/7 is critical for the initiation of the root primordium during de novo root organogenesis [25]. WOX11 and WOX 12 are involved in the first-step cell fate transition during de novo root organogenesis in Arabidopsis [26]. The WOX11-LBD16 pathway promotes pluripotency acquisition in callus cells during de novo shoot regeneration in tissue culture [27]. Deveaux provided evidence in favor of the WOX13 orthologous group as the clade containing the most conserved WOX genes and established a functional link to organ initiation and development in Arabidopsis [28]. It was discovered that WOX13 promotes replum development, likely by preventing the activity of the JAG/FIL genes in medial tissues [29]. WOX4 acts redundantly with WOX14 in the regulation of vascular cell division and organization, playing crucial roles in stem formation [30]. WOX14 overexpression stimulates the expression of GA3ox anabolic genes and represses GA2ox catabolic genes, promoting the accumulation of bioactive GA. These data indicate that WOX14 promotes vascular cell differentiation and lignification in inflorescence stems of Arabidopsis [31].

In addition to their function in plant development, some WOX genes play roles in response to abiotic stress. A mutant allele of AtWOX6 named HOS9-1 is important in freezing tolerance, and comparison between mutant plants and wild plants indicated that the mutant grows more slowly, flowers later and is more sensitive to freezing [20]. In rice, most WOX genes are responsive to abiotic stress stimuli of drought, NaCl and cold [32]. In addition to the study of herbaceous plants, the role of WOX genes in woody plants has also been studied. VvWOX genes appeared to be key regulators of somatic embryogenesis in grapevine [33]. Palovaara’s results provide basic information for studies of the evolution of the WOX gene family and of their function in relation to meristem dynamics and specification of stem cells in gymnosperms [34].

The above results indicate that WOX transcription factors affect plant growth and development mainly by altering the expression of downstream genes. It is noteworthy that the cloning and functional studies of WOX proteins mainly focus on model plants such as A. thaliana and rice. However, there are no comprehensive molecular evolutionary studies on soybean (Glycine max [L.] Merr.). Soybean is one of the most important grain legumes for food, feed and a range of industrial applications worldwide. Soybean has a high nutritional value due to its high protein and oil contents and is a predominant source of isoflavonoids and saponins [35]. Because of the key roles that WOX genes play in various physiological and developmental stages, these genes are potential targets for better and faster growth of soybean. In this study, we identified 33 WOX-encoding genes in soybean and provide a detailed analysis of the expression and function of GmWOXs. Our data will provide a physiological and molecular characterization of soybean to obtain a comprehensive overview of the WOX genes that are potentially involved in soybean developmental processes, including embryonic patterning, stem-cell maintenance, stress response and organ formation. The results of this study provide a good theoretical basis for further research on the potential function of the WOX genes that are involved in soybean growth and development and abiotic stress response.

## 2. Results

### 2.1. Identification of 33 GmWOX Genes and Their Physicochemical Properties

**Figure 1:**
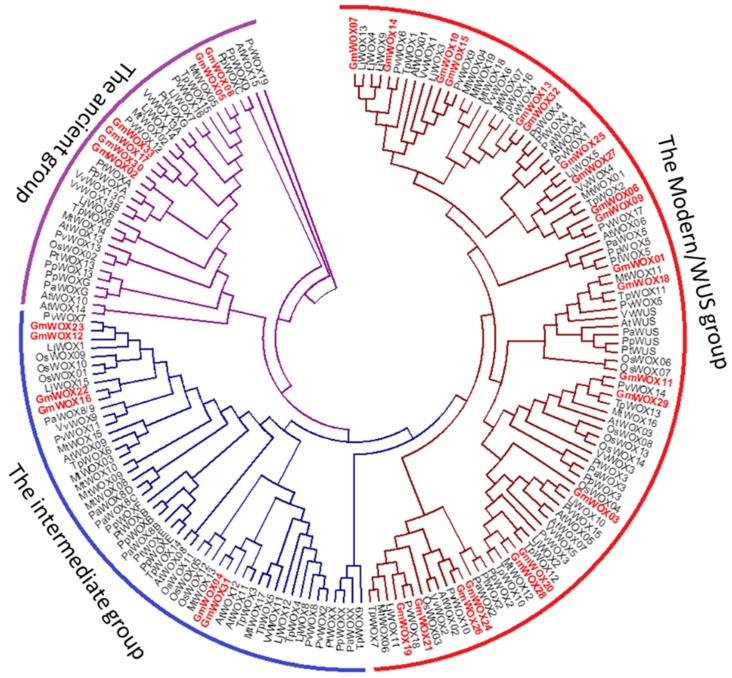
Phylogenetic relationships of the WUSCHEL-related homebox’s (WOXs). Phylogenetic relationships of the WOXs from soybean (Gm), *Medicago truncatula* (Mt), Arabidopsis (At), *Lotus japonicus* (Lj), common bean (Pv), *Trifolium pretense* (Tp), *Picea abies* (Pa), *Pinus pinaster* (Pp), *Vitis vinifera* (Vv) and rice (Os). The phylogenetic tree was constructed using Mega 6. The 181 WOXs from 10 plant species can be divided into 3 groups; branches are colored differently.

To identify WOX family members from soybean, a genome-wide search was carried out using both Hidden Markov Model and BLAST searches. A total of 33 soybean WOX genes were identified in the soybean genome (Table 1), which is nearly twice as many as Arabidopsis (16), rice (14), Lotus japonicus (15), Trifolium pretense (16), Picea abies (12), Pinus pinaster (14) and Vitis vinifera (12) (Figure 1 and Table S1). The 33 WOX genes were named from GmWOX01 to GmWOX33 according to their physical locations (from top to bottom) on chromosomes 1–20 (Table 1).

All of these WOX genes contain the domains PF00046 based on Pfam and SMART tests. Detailed information pertaining to the GmWOX genes is listed in Table 1. The secondary structure of WOX family proteins in soybean was analyzed by SOPMA. The results showed that the secondary structure of these 33 soybean WOX family proteins consisted of four structural forms: alpha heli, extended strand, beta turn and random coil (Table S2). Only the secondary structure of the GmWOX14 protein is mainly alpha-helix, while other GhWOX proteins are mainly random coils.

### 2.2. Phylogenetic Analysis and Structural Predication of the Soybean WOXs

To explore the evolutionary relationship of WOX genes among soybean and other species (Arabidopsis, rice and Medicago), a neighbor-joining (NJ) phylogenetic tree was constructed by MEGA 6.0. A total of 181 WOX genes from soybean (33), Arabidopsis (16), rice (14), Lotus japonicus (15), common bean (19), Trifolium pretense (16), Medicago (19), Picea abies (12), Pinus pinaster (14) and Vitis vinifera (12) were used for phylogenetic analysis (Figure 1). The phylogenetic tree showed that WOX genes could be divided into three major groups: Modern/WUS, intermediate and ancient. The modern/WUS group was the largest group in this phylogenetic tree, which contained 94 members, composed of 21 members from soybean, 7 from rice, 8 from Arabidopsis, 7 from Lotus japonicus, 10 from common bean, 8 from Trifolium pratense, 9 from Medicago, 5 from Picea abies, 5 from Pinus pinaster and 7 from Vitis vinifera. The intermediate group was the second largest group and included 6 soybean, 7 Medicago, 6 rice, 3 Lotus japonicus, 5 common bean, 2 Trifolium pratense, 1 Picea abies, 5 Pinus pinaster, 3 Vitis vinifera and 5 Arabidopsis members. In the ancient group, there were 21 members (6 soybean, 3 Medicago, 1 rice, 3 Lotus japonicus, 3 common bean, 6 Trifolium pratense, 5 Picea abies, 4 Pinus pinaster, 2 Vitis vinifera and 5 Arabidopsis members). Additionally, we also researched the paralogous and orthologous relationships among different plant WOX gene families. These subfamilies included 21 pairs of paralogous genes and 37 pairs of orthologous genes (Figure 1). Some putative orthologs with soybean, namely, LjWOX13/GmWOX07, PvWOX14/GmWOX11, PvWOX06/GmWOX14, TpWOX13/GmWOX29, PvWOX09/GmWOX15, LjWOX10/GmWOX03, PvWOX07/GmWOX23, LjWOX05/GmWOX25, PvWOX17/GmWOX09, PvWOX18/GmWOX21, MtWOX11/GmWOX01 and TpWOX11/GmWOX18 were proposed based on the phylogenetic tree. By analyzing paralogous and orthologous relationships, we also found that most GmWOXs showed closer relationships to PvWOXs. The reason for this might be that Lotus japonicus and soybean both belong to legumes and are more closely related. The analysis of paralogous genes for all the family members confirmed that soybean had undergone two duplication events after the monocot/dicot split, and most of the WOX genes in soybean had expanded in a species-specific manner. We performed a genome-wide comparison of plant WOX members from monocots (rice) and dicots (soybean, Arabidopsis, Lotus japonicus, common bean, Trifolium pratense and Medicago) to explore how the WOX gene family has evolved.

**Figure 2:**
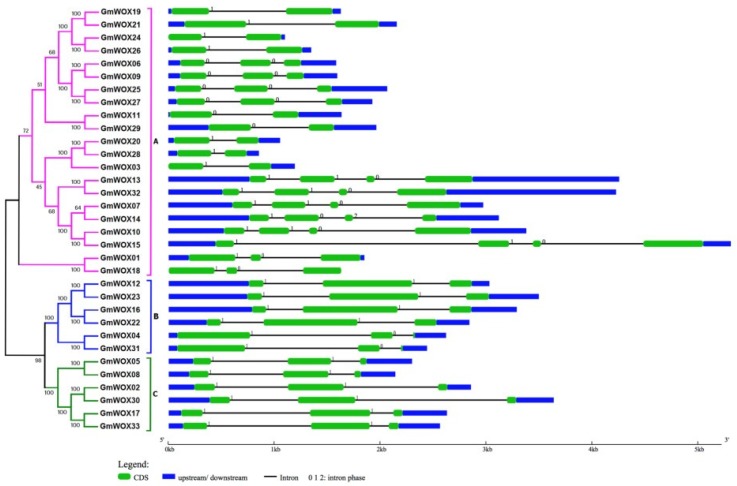
Phylogenetic relationships and gene structures of GmWOXs. The phylogenetic tree (left panel) was constructed using MEGA 6.0, and the gene structures (right panel) were drawn using the Gene Structure Display Server.

Gene structure is important for determining the relationship between genome evolution and the functional divergence of multigene family members. To further examine the structural diversity of the WOX genes in soybean, an exon-intron diagram of the GmWOX genes was constructed according to their genomic and coding sequences by Gene Structure Display Server (GSDS) (Figure 2). The number of introns ranged from one to three in GmWOXs. Combined with the results of phylogenetic trees, we found that group A (modern/WUS) had 1–3 intron insertions. Group A (modern/WUS) was divided into two sub-clades. The first subgroup contained 1 or 3 introns. The second subgroup contained only two members: GmWOX01 and GmWOX18, and they had two introns. Group B (intermediate) and group C (ancient) both contained two introns. There are three types of introns in the GmWOX family. The phase-1 intron (separating the first and second nucleotides of a codon) was found in 27 GmWOXs, the phase-0 intron (interrupting two triplet codons exactly) was found in the 6 remaining WOX genes and only GmWOX14 was found in the phase-2 intron (splitting the second and third nucleotides of a codon). Eighteen members of the GmWOX gene family contain 3 exons, and 6 GmWOX genes possess 4 exons; the other 9 GmWOXs harbor 2 exons (Figure 2). Most of the closely related soybean WOX members in the same subfamilies share similar intron numbers and exon lengths. Additionally, the exon/intron structures of 16 paralogous pair genes were also compared at the terminal branch of the phylogenetic tree to obtain traceable intron gain/loss information. Fourteen of 16 paralogous pairs exhibited conserved exon/intron structures, and only two pairs (GmWOX24/26 and GmWOX01/18) showed certain variations (Figure 2). The different gene structures existing in the different phylogenetic groups suggest that genes evolved into diverse exon-intron structures to carry out different functions in the soybean genome.

### 2.3. Chromosomal Location, Conserved Motifs and Cis-Element Analysis of GmWOXs

**Figure 3:**
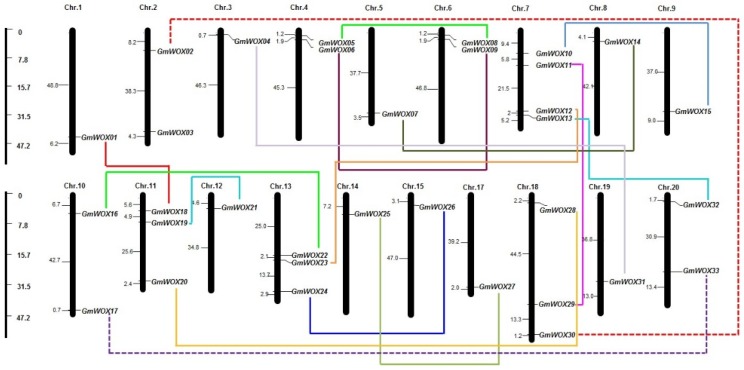
Chromosomal distribution and gene duplications of GmWOXs. The segmental duplicated genes are linked by lines. The scale bar on the left indicates the length (Mb) of soybean chromosomes.

The chromosomal locations and gene structures of all GmWOXs were obtained from the soybean genome database and then realized with a profile diagram constructed by MapDraw V2.1 (Figure 3). The 33 GmWOX genes were widely located on 19 chromosomes except chromosome 16 (Figure 3 and Table 1). Chromosome 7 had the highest density of GmWOX genes, with 4 members; three GmWOX genes were found on chromosomes 11, 13 and 18; two genes were found on chromosomes 2, 4, 6, 10 and 17; and the other chromosomes (1, 3, 5, 8, 9, 12, 14, 15, 17, 19 and 20) only had one GmWOX gene (Figure 3). We investigated gene duplication events to further understand the expansion mechanism of the soybean WOX family. Except for GmWOX03 (Glyma02g42200.1) located outside of a duplicated block, there were 16 pairs of segmental duplications in 32 GmWOX genes (Figure 3 and Table S3), while no pair tandem duplication was found in the GmWOX gene family. Therefore, segmental duplication appeared to have played a crucial role in the expansion of the WOX gene family in soybean.

**Figure 4:**
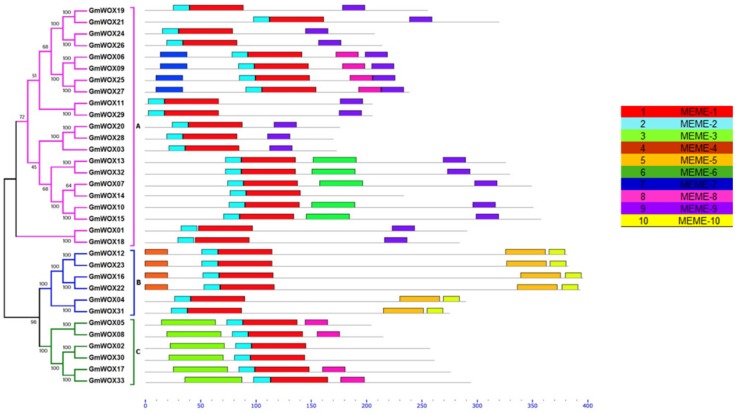
Schematic distribution of the conserved motifs in the soybean WOX family according to MEME. The conserved motifs were identified in the proteins of every group. Each colored box below the tree represents the conserved motifs.

The conserved motifs among GmWOXs were analyzed by MEME suite. A total of 10 conserved motifs were predicted in the GmWOXs (Figure 4). The size of the identified motifs ranged from 15 to 50 amino acids (Table S4). The results showed that each group classified by phylogenetic analysis shared similar conserved motif compositions, however there were some differences between different subgroups. As shown in Figure 4, motifs 1 and 2 were present in all of the GmWOXs. Motif 3 only existed in group C. Motif 4 existed in most of the group B members. Motifs 5 and 10 only existed in group B. Motifs 7 and 8 were present in GmWOX6/9/25/27. Motif 9 was present in all the members of group A. To some extent, these subfamily-specific motifs may lead to the functional differences of WOX genes in soybean. The detailed information is shown in Table S6.

A promoter, as a region of DNA that initiates transcription of a particular gene, plays a prominent role in the temporal and spatial regulation of gene expression. To further understand the biological functions and regulation network that GmWOXs might be involved in, each GmWOX gene was extracted 1.5 kb genomic sequences upstream of its transcriptional start codon for cis-regulatory elements and subjected to analysis in the New PLACE database. The results showed that GmWOX promoter sequences generally carried a variety of predicted cis-acting elements that are responsive to various hormone-related, abiotic stress factors and numerous development-related elements: auxin-responsive elements, abscisic acid-responsive elements, ethylene-responsive element, salicylic acid-responsive elements, gibberellin-responsive elements, jasmonate-responsive elements, abiotic stress-responsive elements and development-related elements. The results are shown in Table S5. However, the pattern of cis-acting elements in promoters of the different GmWOX genes was distinct. Some elements are widely distributed among the promoters of almost all GmWOX genes. GT1GMSCAM4, MYCCONSENSUSAT, GT1CONSENSUS, POLLEN1LELAT52 and WRKY71OS were found in all GmWOX promoters. AGMOTIFNTMYB2 and TELOBOXATEEF1AA1 were only found in GmWOX2, and AGMOTIFNTMYB2 was induced by various stresses, such as wounding or elicitor treatment. MYBATRD22 only existed in GmWOX3, which is a dehydration-responsive element. LTREATLTI78 (putative low temperature-responsive element) was only found in GmWOX5. ATHB6COREAT (hormone responses) existed only in GmWOX19. QARBNEXTA occurs in the promoter region of GmWOX26 and controls activation in response to wounding and tensile stress. Collectively, the element-profiling analysis revealed that GmWOX genes might be widely regulated by a variety of factors, including phytohormones, biotic and abiotic factors and the underlying development regulation factors.

### 2.4. Expression Profile of GmWOXs in Soybean

**Figure 5:**
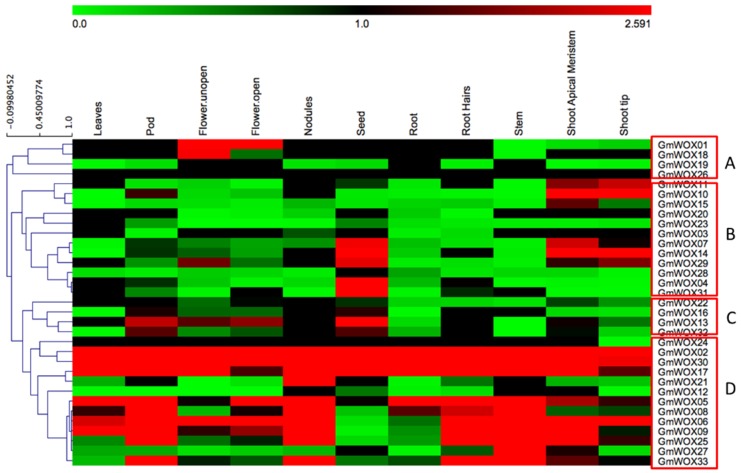
Tissue-specific expression profiles of GmWOX genes. The heatmap shows gene expression patterns of GmWOX genes in 11 different tissues according to Genome Database RNA-Seq data. The color scale above the heat map indicates gene expression levels. The green color indicates a low expression level and the red color indicates a high expression level.

To dissect the expression patterns of GmWOX genes in various tissues, RNA-seq data were downloaded from Phytozome 12. Eleven diverse tissues, i.e., leaves, pods, unopen and open flowers, nodules, seed, roots, root hairs, stems, shoot apical meristems and shoot tips, were included in the analysis (Table S5). We processed the FPKM data and generated a heatmap. From the heat map (Figure 5), we found that the expression patterns of the 33 GmWOX genes were mainly clustered into four groups based on the hierarchical clustering analysis. Group A contained 4 soybean WOX members (GmWOX01, 18, 19, 26) and all these members showed nearly no expression in all 11 tissues. Only GmWOX01 and GmWOX018 were expressed in flowers. Group B included 12 soybean WOX members and most of the members in this group demonstrated no expression or very low expression. Only a few genes were expressed in individual tissues. GmWOX7, GmWOX10, GmWOX11, GmWOX14 and GmWOX15 were highly expressed in the shoot apical meristem. Some gene expressions were tissue specific. GmWOX4, GmWOX7, GmWOX14, GmWOX29 and GmWOX31 showed high expression only in the seed. There were 4 (GmWOX16, GmWOX13, GmWOX22 and GmWOX32) GmWOX members in group C. They were expressed mainly in pods, flowers and seed. There were 13 members in the D group and most of the group members showed consistently high expression in the majority of tissues. Three genes (GmWOX02, GmWOX17 and GmWOX30) showed high expression levels across all of the analyzed tissues. Additionally, 3 soybean WOX genes (GmWOX06, 09 and 25) were clearly upregulated in the stem. These subfamily-specific tissue expression patterns may be closely related to gene functions. The expression patterns of the paralogous pairs were also revealed by heat maps and we found that most of the paralogous pairs with high sequence similarity had similar expression patterns. For example, GmWOX01/18 was only expressed in flowers, with little or no expression in other tissues. Expression divergence was also found in one paralogous pair (GmWOX17/33). GmWOX17 was highly expressed in all tissues, while its paralog, GmWOX33, showed weak expression in six tissues.

**Figure 6:**
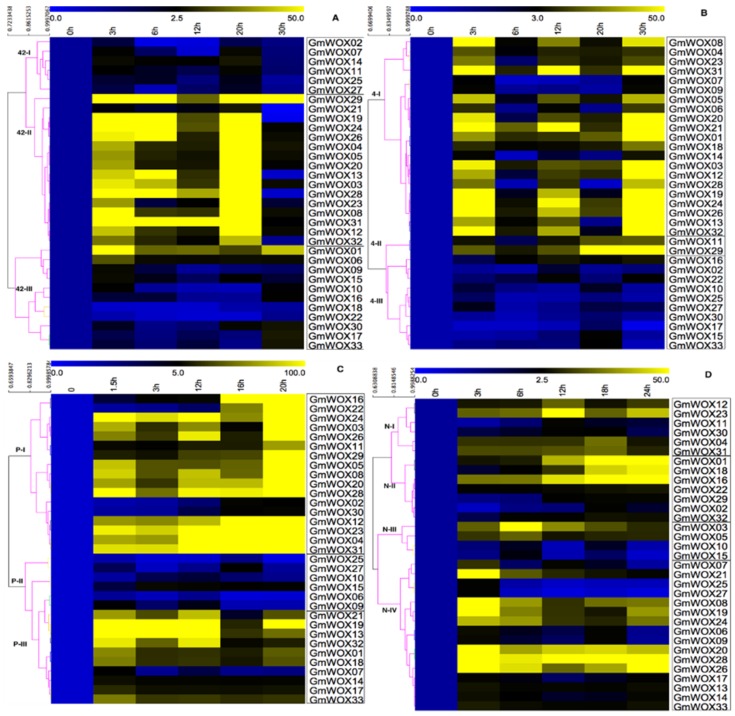
Expression analysis of GmWOX genes in response to abiotic stresses. Two-week-old soybean seedlings were exposed to stress treatment as indicated below. Gene expression analysis was conducted by qRT-PCR using gene-specific primers. (**A**) Heat stress, (**B**) Cold stress, (**C**) Drought stress, (**D**) Salt stress.

To further confirm whether the expression of GmWOX genes was influenced by different abiotic stress treatments, we performed an expression investigation of the 33 WOX genes under drought, heat, cold and salt stress conditions. qRT-PCR experiments were performed to analyze their expression patterns in response to the four abiotic stresses. The results are displayed in Figure 6.

Overall, some GmWOX genes were significantly induced or repressed by multiple treatments. Under heat stress (42 °C), the 33 WOX genes could be roughly divided into three groups of expression patterns (42-I, 42-II and 42-III). Group 42-II members showed high expression, and 42-I and 42-III were upregulated (Figure 6A). For example, group 42-II contained 16 GmWOXs and the members of this group showed obvious upregulation from 3 h to 20 h with peak expression at 20 h (>29.89-fold). Additionally, most of these genes in this group showed strong expression at 3 h and 20 h, while between 6 h and 12 h, they showed a slight decrease. In group 42-I, 6 GmWOX members also showed slightly upregulated expression at 3 h and 20 h, however the highest expression was only a 29.89-fold increase. The 11 GmWOX genes in group 42-III had a low expression similar to group 42-I and most were not highly expressed compared with group II, except GmWOX01. However, the difference was that most members of this group showed the highest expression at 3 h and 20 h, while from 3 h to 20 h, the 11 WOX genes showed a low expression level.

For the cold treatment (Figure 6B), there were four different categories of GmWOX gene expression patterns. Most GmWOX members in groups 4-I and 4-II showed a very significant response to cold compared with 4-III. In addition, the high expression level of GmWOX genes of groups 4-I and 4-II mostly appeared at 3 h, 12 h and 30 h. Most of the GmWOX genes in group 4-III exhibited upregulation, mainly at 20 h. Furthermore, there were 21 GmWOX genes in group 4-I, 2 GmWOX genes in group 4-II and 10 GmWOX genes in group 4-III. In group 4-I, all members were strongly induced at 3 h, 12 h and 30 h, except GmWOX07 and GmWOX09. Both of these genes were only upregulated at 3 h and 30 h and were not obviously expressed between 3 h and 20 h. The genes GmWOX11 and GmWOX29 showed an overall trend towards higher expression under cold conditions, with the exception of the 6 h time point. Compared with the first two groups, the whole of group 4-III showed no obvious response to the cold treatment and only GmWOX16 exhibited a slight reaction between 6 h and 30 h. The other groups showed only a mild reaction at some time point.

Under drought conditions, we observed three main types of expression patterns (Figure 6C): the first group, P-I, which included 17 GmWOXs, showed a general trend toward higher expression under drought stress. The maximal expression level of this group was an increase of approximately fifty times after 20 h of drought stress, except for GmWOX02 and GmWOX30. There were 6 GmWOX genes in the second group, P-II. Compared with the P-I and P-III groups, these genes in P-II were not expressed or downregulated after treatment with 15% PEG. It might be that the genes in this group were not sensitive to drought and may not have been resistant to drought. The last group contained 10 GmWOX genes and showed a very different expression pattern to the other two groups. These genes were enhanced after the early stage under drought treatment and peaked at 1.5 h. As time progressed, the expression level of genes in this group remained high until 20 h, except for GmWOX07. This result suggests that the genes in this group might play an important role in the early stage of stress.

Under salt stress, four groups were clustered (Figure 6D): N-I (6 GmWOX genes), N-II (7 GmWOX genes), N-III (4 GmWOX genes) and N-IV (16 GmWOX genes). The expression patterns of the GmWOX genes during salt treatment exhibited obvious differences, indicating the functional diversity of these genes. The maximum expression levels in the different groups were different; for example, in group N-I, the peak time point appeared between 12 h and 18 h, compared with 18 h to 20 h in group N-II, 6 h in group N-III and 3 h in group N-IV. We also found that in response to salt treatment, some GmWOX genes were obviously enhanced during the whole stage, such as GmWOX20, GmWOX28 and GmWOX26. Additionally, several decreased transcript levels, such as those of GmWOX25 and GmWOX27, were observed.

### 2.5. GmWOXs and Soybean Growth and Development

**Figure 7:**
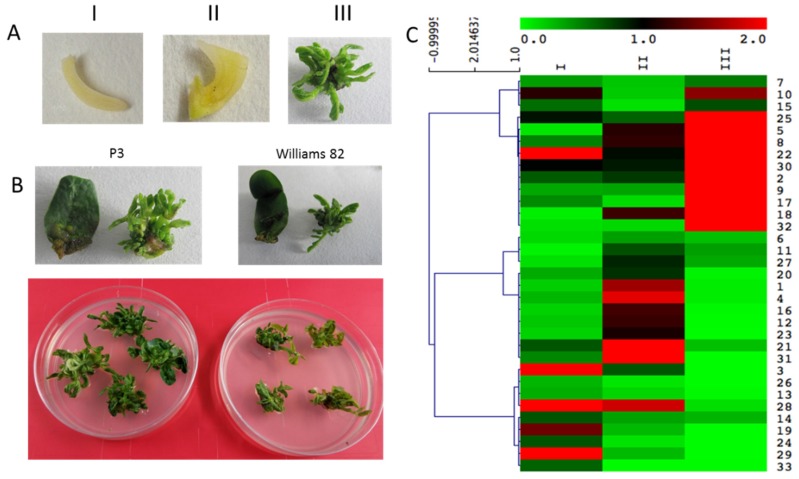
Expression analysis of GmWOX genes in high regeneration capacity soybean material P3. (**A**) Three stages (shoot apical stage, elongated hypocotyl stage, shoot cluster stage) used for sampling; (**B**) Comparison of regeneration ability of two soybean species; (**C**) Expression of GmWOX genes in P3.

Arabidopsis WOXs regulate key developmental processes, including stem cell maintenance of SAM, RAM and CAM, polar patterns at the apex and base of embryos and lateral organ development [6]. The meristem is an important basis for plant regeneration, and the WUS gene plays an important role in stem meristems. Therefore, the expression of the WOX gene can be associated with the plant regeneration ability. To preliminarily understand the function of GmWOXs in soybean regeneration, we performed an expression investigation of the 33 WOX genes in the soybean shoot apical meristem. We compared the expression of the high regeneration capacity soybean material P3 and the ordinary regenerative material Williams 82 (Figure 7B). We selected samples of meristem from three stages (shoot apical, hypocotyl elongate, shoot clusters) (Figure 7A). The qRT-PCR results are shown in Figure 7C. Compared with CK (Williams 82), 4 GmWOXs had significant changes in the expression of the shoot apical, and 4 GmWOXs had significant changes in the expression of the hypocotyl. During adventitious shoot regeneration, 10 GmWOXs had significant changes in expression. The expression levels of GmWOX17, GmWOX18 and GmWOX22 changed more than 150-fold during adventitious shoot regeneration. The findings provide useful information for further elucidation of the functions and mechanisms of WOXs in the development of soybean.

### 2.6. Phenotypes of GmWOX18 Transgenic Soybean

**Figure 8:**
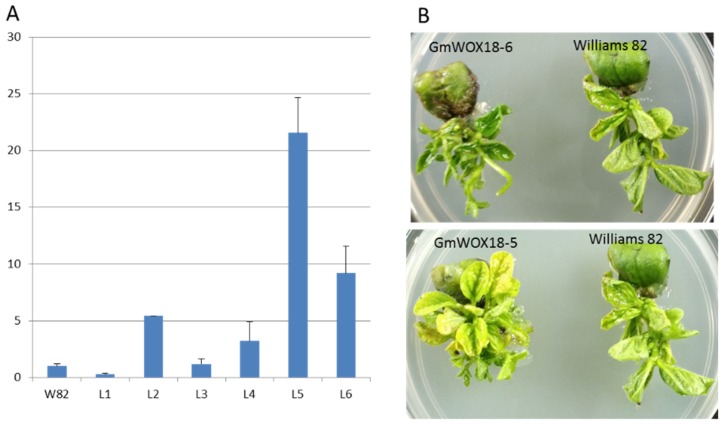
The regeneration ability of GmWOX18 transgenic lines. (**A**) RT-PCR testing of GmWOX18 transgenic lines; (**B**) Induction of cluster buds in transgenic lines.

As described above, the expression of some GmWOX genes was significantly altered by the high regeneration capacity of soybean P3. Combined with the results of evolutionary relationship analysis, we chose GmWOX18 for further functional exploration. Two transgenic lines (GmWOX18-5 and GmWOX18-6) with higher GmWOX18 expression (Figure 8A) were selected for phenotypic analysis. As shown in Figure 8B, the transgenic lines resulted in increased regeneration of tufty buds compared to non-transgenic Williams 82 lines under identical culture conditions. These results revealed that overexpressing GmWOX18 in soybean could increase the regeneration ability of clustered buds. This is of great significance to improve the efficiency of soybean genetic transformation.

## 3. Discussion

### 3.1. Expression and Phylogenetic Analysis of the Soybean WOX Gene Family

Previous analysis of the WOX gene family has been performed in rice, sorghum, maize, Arabidopsis, cotton, poplar, paper mulberry [36], Pinus pinaster [37], grapevine [33], Picea abies [34] and Brassica napus [8,38,39]. However, there are no reports on the analysis of this family in soybean. Thirty-three WOX genes from soybean were identified and analyzed. Through phylogenetic analysis, we also found that the GmWOX gene family can be classified into three clades (modern/WUS, intermediate and ancient) in the neighbor-joining phylogenetic tree. Based on a survey of the existing results, the ancient clade represents the conservative WOXs from lower plant algae to higher plants, the intermediate clade has members from vascular plant species and the modern/WUS clade has members from only spermatophyte species [40]. In Arabidopsis, the functions of most WOX genes have been studied. We compared the homologous genes of soybean and Arabidopsis thaliana (Table S7). We found that members of the ancient clade (WOX10, WOX14 and WOX13) were mainly expressed in roots and inflorescences, and the function is the regulation of root development and fruit development of Arabidopsis [29,31]. GmWOX2/5/8/30/17/33 are members of the ancient clade in soybean. These genes are expressed in most tissues, and the expression quantity is relatively high compared with other clade members. In Arabidopsis, members of the intermediate clade are involved in embryogenesis and morphological development, and members of the modern clade are mainly involved in meristem maintenance [3]. The expression pattern of a gene is often indicative of its functional relevance. Compared to Arabidopsis, we found that the expression between homologous genes was conserved in the modern/WUS clade. Therefore, we speculate that the function of these genes may also be relatively conservative.

### 3.2. WOX Transcription Factors Regulate Plant Growth and Development and Participate in the Abiotic Stress Response

Plant growth is subjected to various environmental stresses during the long-term process of biological evolution. Plants have developed their own resistance to environmental stress through a variety of self-regulatory mechanisms through physiological and metabolic changes in response to stress. Previous studies on WOX genes have mainly focused on plant developmental-related functions, however responses to environmental stresses are seldom known. Previous researchers reported that three types of WOXs (modern/WUS, intermediate and ancient) are exploited to eliminate ROS caused by abiotic stress [8,39]. To further investigate the putative roles of WOX genes in soybean response to abiotic stresses, we examined the expression patterns of 33 soybean genes under heat, cold, drought and salt treatments using qRT-PCR. Our results showed that the GmWOX genes responded to environmental stresses. We found that most of the 16 pairs of duplicated genes (Table S3) exhibited the same expression pattern under different stresses, suggesting that they had the same functions under different stresses. Moreover, we also discovered that some of the duplicated genes, e.g., GmWOX11/29 and GmWOX02/30, were differentially expressed under heat stress; GmWOX11/29, GmWOX02/30, GmWOX13/32 and GmWOX05/08 responded differently when exposed to salt conditions (Figure 6). These results indicate that these pairs of duplicated genes might be the node for multiple stress regulation and suggest that they play different roles under different stresses.

### 3.3. The Function of WOX Genes in Plant Regeneration

Plant shoot apical meristems need to maintain their structural stability during the differentiation of various organs. Stress treatment applied to the soybean cotyledonary node may break the steady state of the meristem structure. Induced by exogenous cytokinins, the expression level increased abnormally. As a result, the number of stem cells increased, and a number of new and complete meristem growth points were established and then developed into clusters of buds. The meristem cells at cotyledonary nodes produce a large number of cluster buds through organogenesis, and stem cells provide primitive cells for the differentiation of cluster buds. WOX genes play an important role in maintaining the stability of the stem tip meristem cell pool [41]. Auxin and cytokinin play critical roles in plant growth and development. WUS induction is regulated by the cytokinin/auxin ratio. Therefore, auxin and cytokinin can trigger the formation of the organizing center and stem cells by inducing WUS and CLV3 expression [42]. Many studies have shown that WUS genes can promote plant regeneration. In Arabidopsis, constitutive expression of WUS resulted in developmentally flexible root cells that could be directed to embryo, leaf or floral organ development, depending on additional cues [43]. Capsicum chinense is similar to soybean and is also a recalcitrant species that is difficult to reproduce by somatic embryogenesis. Introducing the AtWUS gene into C. chinense could promote the transition from vegetative growth to embryogenesis and increase the response to hormones during somatic embryogenesis. After 15 days of induction, the segments of transformed stems began to form globular structures, suggesting that heterologous WUSCHEL was active and involved in the process of morphogenesis [44]. Arroyo-Herrera found that expression of AtWUS in coffee plants induced calli formation as well as a 400% increase in somatic embryo production [45]. The results show that transgenic expression of the transcription factor WUS can be useful to increase somatic embryogenesis in heterologous systems. Here, we report the development of a system for the transformation of soybean Williams 82 using the AtWUS homologous gene GmWOX18 of Glycine max. The results show that overexpression of the transcription factor GmWOX18 can be useful to increase clustered buds in the cotyledon method of genetic transformation. Therefore, overexpression of the WUS gene in some soybean varieties is expected to enhance the regeneration ability of soybean, thereby improving the efficiency of soybean genetic transformation and applying transgenic technology to soybean molecular breeding.

## 4. Materials and Methods

### 4.1. Gene identification

The soybean genome database was used to obtain DNA and protein sequences [46]. To identify all members of the WOX family in soybean, we analyzed the domains of all of the soybean proteins using a Hidden Markov Model (HMM) profile of the WOX domain (PF00011) downloaded from the Pfam 27.0 database [47] with an expected value (e value) cut-off of 0.001. The genome sequences of Arabidopsis thaliana were retrieved from the TAIR database. The genome sequences of Oryza sativa and Medicago were identified from Phytozome 12. The physical and chemical parameters of WOX sequences were predicted by the ExPASy ProtParam tool, including amino acids, molecular weight (d) and pI [48].

### 4.2. Phylogenetic Tree Construction

Phylogenetic and molecular evolutionary analyses were performed by MEGA6 with the neighbor-joining method [49]. The phylogenetic tree contained full-length amino acid sequences of WOX from Arabidopsis, rice, Medicago and soybean. All of the full-length amino acid sequences were aligned with ClustalX using default parameters [50]. The soybean WOX genes were classified into different groups according to the topology of the phylogenetic tree and the classifications of WOXs in four other species.

### 4.3. GmWOX Gene Structure and Conserved Motif

GmWOXs exon/intron structures were analyzed using the gene structure display server 2.0 [51]. The conserved motifs were predicted by the MEME program (http://alternate.meme-suite.org/tools/meme) [52], with the following parameters: any number of repetitions, maximum of 10 misfits and an optimum motif width of 6–200 amino acid residues.

### 4.4. Chromosomal Location and Gene Duplication

The chromosomal locations, intron numbers and sizes (bp) of soybean WOX genes were obtained using Phytozome 12 [46]. The chromosomal locations of the WOX genes were presented using MapDraw V2.1 software [53]. Gene duplication was confirmed based on the length of the shorter aligned sequence that covered >70% of the longer sequence, and the similarity of the two aligned sequences was >70%. Two genes separated by five or fewer genes in the 100 kb chromosome fragment were considered tandem duplicated genes. Segmental duplications were identified by synteny analysis using an online tool of the Plant Genome Duplication Database (PGDD) [53].

### 4.5. Tissue-Specific Expression Profile of GmWOXs

For tissue-specific expression profile analysis, the transcription data of WOX gene expression in soybean were downloaded from Phytozome 12. The genome database data included various tissues (leaves, pods, unopen and open flowers, nodules, seed, roots, root hairs, stems, shoot apical meristems and shoot tips). The expression data were gene-wise normalized and the heatmap was drawn using MeV4.9 software [54]. Hierarchical clustering analysis was conducted using clustering distance ‘‘correlation’’ (Pearson correlation), and the clustering method used was ‘‘complete’’ (complete linkage method).

### 4.6. Plant Materials and Treatments

The soybean seeds of Williams 82 were germinated in water at 25 °C under dark conditions, and seedlings were subjected to a 12 h light and 12 h dark photoperiod and 70% humidity. Stress treatments were applied to 2-week-old soybean seedlings.

For heat stress, the soybean seedlings were exposed to 42 ± 2 °C; for cold stress, the seedlings were exposed to a temperature of 4 ± 2 °C; for salt stress, the seedlings were incubated with 150 mM NaCl; and for drought stress, the seedlings were treated with 15% PEG. After different time points, whole plants were collected. Then, all of the collected samples were rapidly frozen in liquid nitrogen and stored at −80 °C for RNA extraction. Three biological replications were carried out.

Total RNA was isolated using the TRIzol reagent following the supplier’s instructions (Transgen, Beijing, China). cDNA was reverse transcribed by TransScript first-strand complementary DNA (cDNA) synthesis SuperMix (Transgen). qRT-PCR was carried out using the TransScript tip green qPCR SuperMix kit, following the manufacturer’s instructions (Transgen). The Glyma.19G147900.1 gene was used as an internal control [55]. Statistical analyses were performed using a t-test, and *p* < 0.05 and < 0.01 were considered significant and extremely significant differences, respectively.

### 4.7. Overexpression of GmWOX18 in Soybean

The soybean seeds of Willams 82 and P3 were surface sterile with chlorine and were germinated overnight in water at 25 °C under dark conditions. Seed coats and embryo roots were removed, and the seed was cut along the hypocotyl into two cotyledons with a cotyledonary node. The cotyledons with a cotyledonary node were then used as explants.

The explants were placed in Φ 9 cm plates containing MS medium with exogenous cytokinin 6-BA 1.67 mg/L at 25 °C under a 16/8 h photoperiod at 140 µmol/s/m^2^, with 5 explants in each plate. The medium was refreshed every two weeks. The cultured cotyledonary nodes at 0, 7 and 22 days were collected for expression analysis. All of the collected samples were rapidly frozen in liquid nitrogen and stored at −80 °C for RNA extraction. Three biological replications were carried out.

The full-length coding sequence (using the primers 5′ ATGATGGAACCTCAACAACAACAA 3′ and 5′ TTAATCATAATCTGGAGACCTGCG 3′) of GmWOX18 was cloned into the XbaI/SacI site of the pTF101 vector. The recombinant construct containing the 2x35S::GmWOX18 cassette was introduced into Agrobacterium tumefaciens strain EHA105 and then transformed into soybean Willams 82 using the Agrobacterium rhizogene-mediated cotyledonary node method [56].

## Figures and Tables

**Table 1:** GmWOX gene information.

Gene Name	Genome ID	Chromosome	Exons/Introns	Transcript Length (bp)	Amino Acid Length	Molecular Weight (Da)	pI
GmWOX01	Glyma01g37190.2	Chr01	3/2	1110	296	33,266.52	7.13
GmWOX02	Glyma02g10410.1	Chr02	3/2	1241	262	29,686.27	5.91
GmWOX03	Glyma02g42200.1	Chr02	2/2	753	177	20,452.13	9.1
GmWOX04	Glyma03g01000.1	Chr03	3/2	1253	295	32,100.75	6.63
GmWOX05	Glyma04g01830.1	Chr04	3/2	1268	208	23,564.8	6.44
GmWOX06	Glyma04g04310.1	Chr04	3/2	1110	224	25,896.42	9.26
GmWOX07	Glyma05g33850.1	Chr05	4/3	1879	357	40,718.48	8.87
GmWOX08	Glyma06g01940.1	Chr06	3/2	1162	219	24,937.32	6.17
GmWOX09	Glyma06g04470.1	Chr06	3/2	1111	230	26,599.11	9.08
GmWOX10	Glyma07g11372.1	Chr07	4/3	2101	357	40,518.28	6.81
GmWOX11	Glyma07g15710.2	Chr07	2/1	1045	210	24,174.19	9.1
GmWOX12	Glyma07g32430.1	Chr07	3/2	1649	388	43,124.1	7.74
GmWOX13	Glyma07g34425.1	Chr07	4/3	2071	333	37,234.08	6.67
GmWOX14	Glyma08g05831.1	Chr08	4/3	2044	238	27,673.2	10.07
GmWOX15	Glyma09g30831.1	Chr09	4/3	1787	364	40,892.7	7.81
GmWOX16	Glyma10g08030.1	Chr10	3/2	2062	403	43,911.23	8.65
GmWOX17	Glyma10g43580.2	Chr10	3/2	1361	281	31,687.29	6.15
GmWOX18	Glyma11g08091.1	Chr11	3/2	870	289	32,527.58	7.8
GmWOX19	Glyma11g14940.2	Chr11	2/1	889	261	29,224.34	5.66
GmWOX20	Glyma11g34990.1	Chr11	2/1	1746	400	43,763.14	8.8
GmWOX21	Glyma12g06895.1	Chr12	2/1	1297	327	36,678.04	6.35
GmWOX22	Glyma13g21860.2	Chr13	3/2	791	180	20,575.94	6.32
GmWOX23	Glyma13g24150.1	Chr13	3/2	1747	389	43,249.12	6.99
GmWOX24	Glyma13g41000.1	Chr13	2/1	673	212	24,109.08	8.93
GmWOX25	Glyma14g09310.1	Chr14	3/2	1270	231	26,520.89	9.03
GmWOX26	Glyma15g04460.1	Chr15	2/1	771	219	24,907.86	7.66
GmWOX27	Glyma17g35880.2	Chr17	3/2	1091	244	28,079.58	9.38
GmWOX28	Glyma18g03350.1	Chr18	2/1	718	174	20,085.46	6.83
GmWOX29	Glyma18g39520.2	Chr18	2/1	1397	210	24,182.08	8.96
GmWOX30	Glyma18g52491.1	Chr18	3/2	1433	266	30,073.53	5.58
GmWOX31	Glyma19g29660.2	Chr19	3/2	1143	280	30,294.91	7.06
GmWOX32	Glyma20g02161.1	Chr20	4/3	1920	337	37,938.99	7.21
GmWOX33	Glyma20g23220.2	Chr20	3/2	1365	284	32,167.86	6.46

## Supplementary Materials

The following are available online at https://www.mdpi.com/2223-7747/8/7/215/s1, Figure S1: Prediction of secondary structure of GmWOX family gene proteins. The helices, turns and coils are indicated as long, intermediate and short vertical lines, respectively. Table S1: Accession numbers of WOX genes from different plants used in the neighbor-joining phylogenetic tree. Table S2: Prediction of secondary structure and subcellular localization of GmWOX family gene proteins. Table S3: Pairwise identities between paralogous pairs of WOX genes from soybean. Table S4: Different motifs commonly observed in soybean protein sequences by the MEME server. Table S5: The transcription data of WOX gene expression in soybean. Table S6: Number of cis-acting elements in 33 GmWOXs. Table S7: Summary of WOX protein expression domains and function in soybean and Arabidopsis.
