# Supplementary material for: Genome-Wide Analysis of the WOX Gene Family and Function Exploration of GmWOX18 in Soybean
Source: Plants (Basel). 2019 Jul 11;8(7):215. doi: 10.3390/plants8070215 (PMC6681341; doi:10.3390/plants8070215)
Supplement: Supplementary file 1 [file plants-08-00215-s001.zip › Supplementary/Table S2.docx]

Table S2 Prediction of secondary structure and subcellular localization for GmWOX family genes protein

| Gene name | Alpha helix% | Extended strand | Beta turn | Random coil | Protein localization |
| --- | --- | --- | --- | --- | --- |
| GmWOX01 | 20.95 | 8.11 | 2.70 | 68.24 | nucl |
| GmWOX02 | 34.35 | 6.49 | 2.67 | 56.49 | nucl |
| GmWOX03 | 38.98 | 12.43 | 6.21 | 42.37 | nucl |
| GmWOX04 | 17.63 | 23.05 | 8.81 | 50.51 | nucl |
| GmWOX05 | 38.46 | 8.17 | 4.81 | 48.56 | nucl |
| GmWOX06 | 28.57 | 6.25 | 2.68 | 62.50 | nucl |
| GmWOX07 | 26.61 | 14.29 | 2.80 | 56.30 | nucl |
| GmWOX08 | 37.44 | 5.94 | 5.02 | 51.60 | nucl |
| GmWOX09 | 29.13 | 8.70 | 4.78 | 57.39 | nucl |
| GmWOX10 | 23.81 | 13.45 | 2.24 | 60.50 | nucl |
| GmWOX11 | 32.38 | 10.00 | 2.38 | 55.24 | nucl |
| GmWOX12 | 13.66 | 14.69 | 3.87 | 67.78 | nucl |
| GmWOX13 | 22.52 | 9.61 | 3.30 | 64.56 | nucl |
| GmWOX14 | 50.00 | 7.14 | 6.30 | 36.55 | nucl |
| GmWOX15 | 25.55 | 12.64 | 2.47 | 59.34 | nucl |
| GmWOX16 | 14.39 | 15.14 | 4.47 | 66.00 | nucl |
| GmWOX17 | 28.83 | 4.27 | 3.20 | 63.70 | nucl |
| GmWOX18 | 19.03 | 6.92 | 2.77 | 71.28 | nucl |
| GmWOX19 | 18.01 | 11.11 | 5.36 | 65.52 | nucl |
| GmWOX20 | 26.67 | 13.89 | 6.11 | 53.33 | nucl |
| GmWOX21 | 19.88 | 16.82 | 6.12 | 57.19 | nucl |
| GmWOX22 | 13.50 | 14.25 | 5.50 | 66.75 | nucl |
| GmWOX23 | 12.85 | 13.37 | 3.34 | 70.44 | nucl |
| GmWOX24 | 20.28 | 9.91 | 2.83 | 66.98 | nucl |
| GmWOX25 | 27.27 | 7.79 | 3.03 | 61.90 | nucl |
| GmWOX26 | 18.72 | 13.24 | 4.11 | 63.93 | nucl |
| GmWOX27 | 28.69 | 7.79 | 2.87 | 60.66 | nucl |
| GmWOX28 | 36.78 | 12.64 | 6.90 | 43.68 | nucl |
| GmWOX29 | 27.14 | 10.00 | 2.86 | 60.00 | nucl |
| GmWOX30 | 30.83 | 6.77 | 3.01 | 59.40 | nucl |
| GmWOX31 | 17.86 | 15.00 | 7.14 | 60.00 | nucl |
| GmWOX32 | 24.93 | 12.46 | 2.67 | 59.94 | nucl |
| GmWOX33 | 32.04 | 9.86 | 3.52 | 54.58 | nucl |
