# Supplementary material for: Genome-Wide Analysis of the WOX Gene Family and Function Exploration of GmWOX18 in Soybean
Source: Plants (Basel). 2019 Jul 11;8(7):215. doi: 10.3390/plants8070215 (PMC6681341; doi:10.3390/plants8070215)
Supplement: Supplementary file 1 [file plants-08-00215-s001.zip › Supplementary/Table S6.docx]

| Regulator | *Cis*-actingelement | Signal sequence | Number of elements in 33 GmWOXs | | | | | | | | | | | | | | | | | | | | | | | | | | | | | | | | |
| --- | --- | --- | --- | --- | --- | --- | --- | --- | --- | --- | --- | --- | --- | --- | --- | --- | --- | --- | --- | --- | --- | --- | --- | --- | --- | --- | --- | --- | --- | --- | --- | --- | --- | --- | --- |
|  |  |  | 1 | 2 | 3 | 4 | 5 | 6 | 7 | 8 | 9 | 10 | 11 | 12 | 13 | 14 | 15 | 16 | 17 | 18 | 19 | 20 | 21 | 22 | 23 | 24 | 25 | 26 | 27 | 28 | 29 | 30 | 31 | 32 | 33 |
| abiotic stress  auxin  ABA  abiotic stress  Ethylene  SA  abiotic stress  abiotic stress  abiotic stress  auxin  development  GA  GA  ABA  ABA  auxin  SA  GA  abiotic stress  stress  abiotic stress  GA  abiotic stress  abiotic stress  ABA  abiotic stress  GA  abiotic stress  ABA  development  development  auxin  abiotic stress  ABA  JA  development  abiotic stress  abiotic stress  abiotic stress  abiotic stress  development  development  Auxin  abiotic stress  gibberellin  gibberellin  auxin  development  hormone  ABA  JA  abiotic stress  ABA | ACGTATERD1  ARFAT  DPBFCOREDCDC3  ELRECOREPCRP1  ERELEE4  GT1CONSENSUS  GT1GMSCAM4  MYB1AT  MYCCONSENSUSAT  NTBBF1ARROLB  POLLEN1LELAT52  PYRIMIDINEBOXHVEPB1  PYRIMIDINEBOXOSRAMY1A  RYREPEATBNNAPA  RYREPEATVFLEB4  SURECOREATSULTR11  WBOXATNPR1  WRKY71OS  ABRELATERD1  AGMOTIFNTMYB2  ASF1MOTIFCAMV  GAREAT  LTRE1HVBLT49  MYB2AT  MYB2CONSENSUSAT  MYBCORE  MYBGAHV  MYCATERD1  MYCATRD22  TELOBOXATEEF1AA1  WUSATAg  CATATGGMSAUR  CBFHV  MYBATRD22  T/GBOXATPIN2  AT1BOX  DRE2COREZMRAB17  DRECRTCOREAT  LTREATLTI78  LTRECOREATCOR15  XYLAT  L1BOXATPDF1  AUXREPSIAA4  NRRBNEXTA  GARE1OSREP1  GARE2OSREP1  BOXCPSAS1  LEAFYATAG  ATHB6COREAT  GBOXLERBCS  GCCCORE  QARBNEXTA  ABREATRD22 | ACGT  TGTCTC  ACACNNG  TTGACC  AWTTCAAA  GRWAAW  GAAAAA  WAACCA  CANNTG  ACTTTA  AGAAA  TTTTTTCC  CCTTTT  CATGCA  CATGCATG  GAGAC  TTGAC  TGAC  ACGTG  AGATCCAA  TGACG  TAACAAR  CCGAAA  TAACTG  YAACKG  CNGTTR  TAACAAA  CATGTG  CACATG  AAACCCTAA  TTAATGG  CATATG  RYCGAC  CTAACCA  AACGTG  AATATTTTTATT  ACCGAC  RCCGAC  ACCGACA  CCGAC  ACAAAGAA  TAAATGYA  KGTCCCAT  TAGTGGAT  TAACAGA  TAACGTA  CTCCCAC  CCAATGT  CAATTATTA  MCACGTGGC  GCCGCC  AACGTGT  RYACGTGGYR | 4  1  2  2  1  16  7  1  2  3  12  1  3  4  2  1  3  8  0  0  0  0  0  0  0  1  0  0  0  0  0  0  0  0  0  0  0  0  0  0  0  0  0  0  0  0  0  0  0  0  0  0  0 | 2  1  2  0  2  20  6  2  18  3  14  1  3  0  0  1  3  9  1  1  1  1  1  1  2  2  1  1  1  1  1  0  0  0  0  0  0  0  0  0  0  0  0  0  0  0  0  0  0  0  0  0  0 | 4  0  0  0  0  17  6  4  6  4  7  0  1  0  0  0  0  2  1  0  0  3  0  2  3  5  3  2  2  0  0  2  1  1  1  0  0  0  0  0  0  0  0  0  0  0  0  0  0  0  0  0  0 | 6  1  1  2  0  9  1  3  0  4  11  0  0  0  0  3  2  5  1  0  0  2  0  0  2  1  1  0  0  0  1  0  0  0  0  1  0  0  0  0  0  0  0  0  0  0  0  0  0  0  0  0  0 | 2  1  2  1  0  15  4  5  6  3  8  0  2  0  0  2  2  7  0  0  0  0  0  0  0  1  0  1  1  0  0  2  2  0  0  0  1  1  1  2  1  0  0  0  0  0  0  0  0  0  0  0  0 | 10  0  2  0  0  13  4  0  2  4  7  0  2  1  0  0  3  5  1  0  2  1  2  0  0  1  1  0  0  0  1  0  0  0  0  0  0  0  0  0  0  1  0  0  0  0  0  0  0  0  0  0  0 | 6  1  0  0  0  11  2  3  6  0  13  0  3  6  2  2  3  10  2  0  1  0  0  0  1  3  0  0  0  0  0  2  0  0  0  0  0  0  0  0  1  0  1  1  0  0  0  0  0  0  0  0  0 | 2  1  2  0  1  13  6  1  4  2  8  0  1  0  0  4  2  5  0  0  0  3  0  0  1  1  3  1  1  0  1  0  0  0  0  0  0  0  0  0  0  0  0  1  0  0  0  0  0  0  0  0  0 | 6  0  1  0  0  20  6  0  10  6  12  0  1  1  0  0  3  3  0  0  1  2  1  0  0  2  1  2  2  0  2  2  0  0  0  0  0  0  0  0  0  1  0  0  2  1  0  0  0  0  0  0  0 | 10  4  1  1  1  15  7  0  2  3  15  0  7  2  0  6  2  7  2  0  0  1  0  0  0  0  1  1  1  0  0  0  0  0  1  0  0  0  0  0  2  0  1  0  0  0  1  0  0  0  0  0  0 | 2  1  2  1  0  15  7  1  14  2  11  0  3  4  2  3  3  7  1  0  0  1  0  0  0  2  0  1  1  0  0  2  0  0  0  0  0  0  0  0  0  0  0  0  0  0  0  0  0  0  0  0  0 | 0  0  2  0  0  12  1  2  8  4  13  0  2  2  0  1  5  14  0  0  1  2  1  1  2  3  2  1  1  0  1  0  0  0  0  0  0  0  0  0  0  0  0  0  0  0  0  0  0  0  0  0  0 | 6  1  2  1  0  10  4  3  10  3  14  2  4  0  0  2  4  8  1  0  0  1  0  3  5  7  0  1  1  0  0  4  0  0  0  0  0  0  0  0  0  0  0  0  0  0  0  1  0  0  0  0  0 | 6  2  0  0  0  10  1  3  4  0  10  0  3  6  0  3  2  8  2  0  1  0  0  0  0  2  0  0  0  0  1  2  0  0  0  0  0  0  0  0  0  0  1  0  1  0  0  0  0  0  0  0  0 | 6  4  1  1  1  17  7  0  10  1  6  2  6  1  0  6  2  5  1  0  2  1  0  0  1  4  1  1  1  0  0  0  1  0  1  0  1  1  0  1  2  0  0  0  0  0  1  0  0  0  0  0  0 | 4  3  2  0  0  22  6  0  18  1  18  2  4  0  0  3  4  5  2  0  0  0  1  1  1  3  0  0  0  0  0  2  1  0  1  0  0  0  0  0  0  2  0  0  0  0  0  0  0  0  0  0  0 | 10  0  1  0  0  10  3  2  4  3  11  0  0  0  0  1  3  7  1  0  0  1  0  3  3  3  0  2  2  0  0  0  1  0  0  0  0  0  0  0  0  0  0  0  0  0  0  0  0  0  0  0  0 | 4  0  1  0  1  15  6  2  2  1  14  0  3  3  0  1  0  7  0  0  0  0  0  1  1  1  0  0  0  0  0  0  0  0  0  0  0  0  0  0  0  1  0  0  0  0  0  0  0  0  0  0  0 | 10  0  1  0  1  11  7  0  12  0  14  1  1  3  0  0  2  10  1  0  1  1  0  1  1  7  0  1  1  0  0  2  0  0  1  0  0  0  0  0  0  0  0  1  1  2  0  1  1  0  0  0  0 | 6  1  2  0  2  13  3  4  10  4  10  0  4  1  0  2  2  5  4  0  0  1  0  0  1  2  1  1  1  0  0  0  0  0  1  0  0  0  0  0  0  1  0  0  1  0  0  0  0  1  0  0  1 | 18  1  1  0  1  16  6  0  10  0  18  0  0  2  0  1  4  15  2  0  1  1  0  1  1  5  0  1  1  0  1  2  0  0  1  0  0  0  0  0  0  0  0  0  1  3  0  2  0  0  0  0  0 | 0  3  2  0  2  15  3  5  18  4  16  0  6  0  0  3  2  5  0  0  1  1  1  1  3  6  1  0  0  0  0  2  0  0  0  0  0  0  0  0  1  1  0  0  0  0  0  1  0  0  0  0  0 | 4  1  2  1  0  13  6  1  12  2  15  1  3  2  0  3  7  11  1  0  0  0  1  2  2  5  0  0  0  0  0  2  0  0  0  0  0  0  0  0  0  0  0  0  1  1  1  0  0  0  0  0  0 | 2  1  1  1  1  16  5  1  10  3  13  1  2  2  0  1  4  6  0  0  0  1  0  0  1  1  0  0  0  0  0  4  0  0  0  0  0  0  0  1  0  0  0  0  0  0  0  0  0  0  1  0  0 | 4  0  1  0  0  19  6  0  10  4  10  1  6  0  0  1  3  5  1  0  1  1  1  1  3  4  1  0  0  0  0  6  0  0  0  0  0  0  0  1  0  0  0  0  0  0  0  0  0  0  0  0  1 | 2  0  0  0  0  11  4  3  10  3  9  0  0  5  4  2  1  3  1  0  0  2  0  0  1  2  2  0  0  0  0  0  0  0  1  0  0  0  0  0  0  0  0  0  0  0  0  0  0  0  1  1  0 | 4  1  1  0  1  11  4  3  8  3  5  0  2  0  0  2  3  6  1  0  1  1  1  0  1  3  1  1  1  0  0  2  2  0  0  1  0  0  0  1  0  0  0  0  0  0  0  0  0  0  0  0  1 | 6  1  2  0  1  14  3  4  10  6  8  1  3  0  0  2  2  6  3  0  0  0  0  0  0  0  0  1  1  0  1  0  0  0  0  0  0  0  0  0  0  0  0  0  0  0  0  0  0  0  0  0  1 | 4  2  1  1  2  14  3  1  18  1  12  0  4  2  0  6  3  7  2  0  0  0  0  1  3  6  0  3  3  0  0  0  0  0  1  0  0  0  0  0  0  0  0  0  1  0  0  0  0  0  0  0  0 | 4  2  3  0  0  13  3  1  12  2  10  1  1  2  0  3  2  9  4  0  0  3  2  0  1  4  2  1  1  1  0  0  2  0  0  0  0  1  0  1  0  0  0  1  0  0  0  1  0  0  1  0  0 | 2  0  4  0  1  12  1  4  4  1  10  0  1  1  0  1  2  6  0  0  0  0  0  0  2  2  0  1  1  0  0  0  0  0  0  0  0  0  0  0  0  0  0  0  0  0  0  0  0  0  0  0  0 | 6  2  1  1  0  13  5  5  12  3  13  3  2  0  0  2  3  9  1  0  0  2  0  1  2  3  1  2  2  0  1  2  0  0  0  0  0  0  0  0  0  1  0  0  0  0  0  2  0  0  0  0  0 | 6  1  0  0  1  18  5  0  6  2  16  0  1  0  0  2  0  5  1  0  1  0  0  0  0  2  0  2  2  0  0  0  0  0  0  0  0  0  0  0  0  0  0  0  0  0  0  0  0  0  0  0  0 |
