# Supplementary figures and images for: Genome-Wide Analysis of the WOX Gene Family and Function Exploration of GmWOX18 in Soybean
Source: Plants (Basel). 2019 Jul 11;8(7):215. doi: 10.3390/plants8070215 (PMC6681341; doi:10.3390/plants8070215)

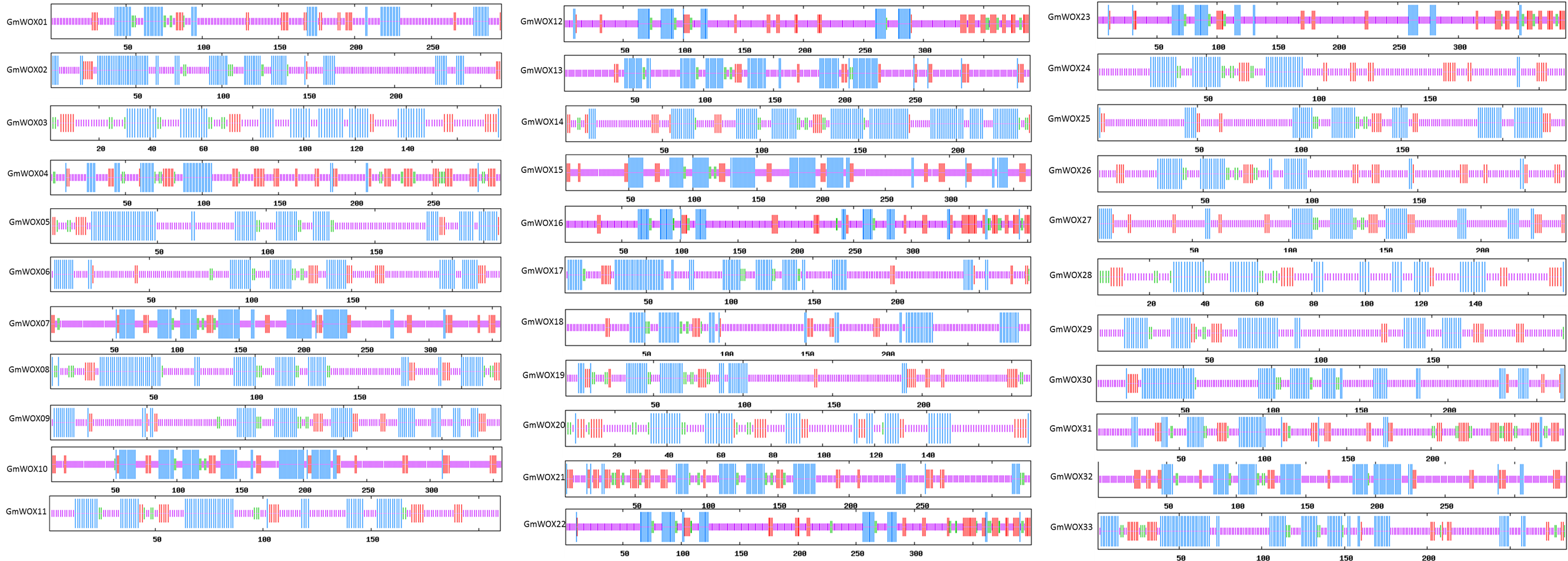

Supplement: Supplementary file 1 [file plants-08-00215-s001.zip › Supplementary/Fig S1.tif]
